# Modulation of Osteoclast Interactions with Orthopaedic Biomaterials

**DOI:** 10.3390/jfb9010018

**Published:** 2018-02-26

**Authors:** Chris Steffi, Zhilong Shi, Chee Hoe Kong, Wilson Wang

**Affiliations:** Department of Orthopaedic Surgery, Yong Loo Lin School of Medicine, National University of Singapore, NUHS Tower Block, Level 11, 1E Kent Ridge Road, Singapore 119228, Singapore; chrissteffi@u.nus.edu (C.S.); dossz@nus.edu.sg (Z.S.); e0012328@u.nus.edu (C.H.K.)

**Keywords:** osteoclasts, monocytes, osteoblasts, implants, polymers and scaffolds

## Abstract

Biomaterial integration in bone depends on bone remodelling at the bone-implant interface. Optimal balance of bone resorption by osteoclasts and bone deposition by osteoblasts is crucial for successful implantation, especially in orthopaedic surgery. Most studies examined osteoblast differentiation on biomaterials, yet few research has been conducted to explore the effect of different orthopaedic implants on osteoclast development. This review covers, in detail, the biology of osteoclasts, in vitro models of osteoclasts, and modulation of osteoclast activity by different implant surfaces, bio-ceramics, and polymers. Studies show that surface topography influence osteoclastogenesis. For instance, metal implants with rough surfaces enhanced osteoclast activity, while smooth surfaces resulted in poor osteoclast differentiation. In addition, surface modification of implants with anti-osteoporotic drug further decreased osteoclast activity. In bioceramics, osteoclast development depended on different chemical compositions. Strontium-incorporated bioceramics decreased osteoclast development, whereas higher concentrations of silica enhanced osteoclast activity. Differences between natural and synthetic polymers also modulated osteoclastogenesis. Physiochemical properties of implants affect osteoclast activity. Hence, understanding osteoclast biology and its response to the natural microarchitecture of bone are indispensable to design suitable implant interfaces and scaffolds, which will stimulate osteoclasts in ways similar to that of native bone.

## 1. Bone Biology of Osteoclasts

### 1.1. Bone Remodelling

Bone is a vastly dynamic tissue, which goes through remodelling during the course of life, even if it seems static. Bone constantly adapts itself to various loads. The phenomenon of adaptation of bone structure to various loads, or rather bone remodelling, was explained by Julius Wolff and is known as Wolff’s Law [1]. For decades, bone remodelling has been investigated and explained from the macroscopic tissue level to microscopic cellular/molecular level. Bone homeostasis at macroscopic level is termed as bone turn over and the turn-over rate for adult human bone is about 10% per annum [2], whereas, the cellular mechanism causing bone turn-over is called as bone remodelling [2]. Constant bone remodelling, the physiological process of replacing old or damaged bone with new bone tissue, contributes an imperative role in scar-free bone healing and regeneration of damaged bone, while maintaining mineral homeostasis [3,4,5,6,7]. Bone remodelling is performed mainly via the coordinated activities between bone-forming osteoblasts and bone-resorbing osteoclasts. The functional and anatomical site of bone remodelling will contain bone cells, including osteoblasts, osteoclasts, bone-lining cells, and osteocytes, that form a basic multicellular unit (BMU). In addition to these cells, immune cells including macrophages, B-cells, and T-cells are also involved in bone remodelling [8].

**Figure 1:**
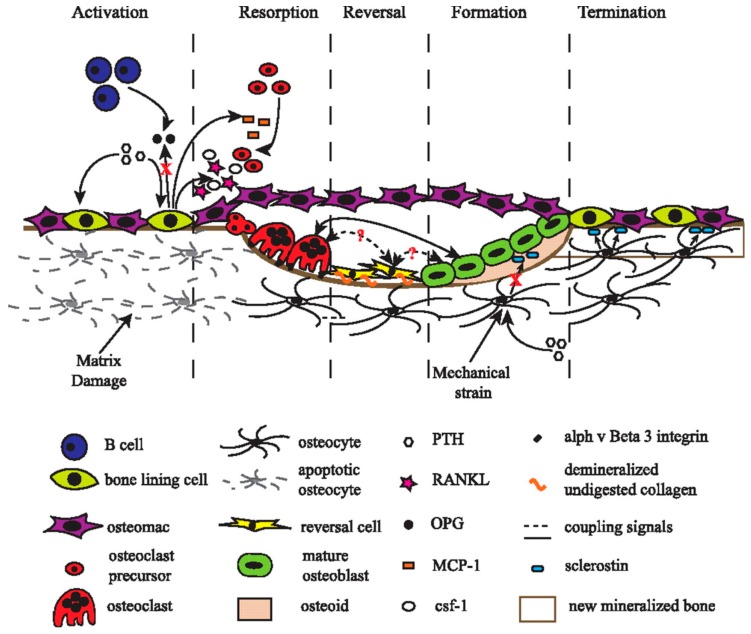
A schematic representation of different stages of bone remodelling. B cells in the marrow of resting bone secrete osteoprotegerin (OPG), which controls osteoclast activation. *Activation phase*: Damaged bone matrix triggers osteocyte apoptosis. Parathyroid (PTH) activates bone cells to recruit osteoclast precursors. *Resorption phase*: monocyte chemoattractant protein-1 (MCP-1) secreted by osteoblasts attracts osteoclast precursors to the damaged site. Osteoblasts also secrete Receptor Activator of NF-κB ligand (RANKL) and colony stimulating factor-1 (CSF-1), which lead to proliferation and differentiation of osteoclast precursors. Functionally-mature osteoclasts attach to the surface, form a sealing zone, and resorb damaged matrix. *Reversal phase*: matrix debris, such as undigested collagen, are removed by reversal cells. Coupling signals generated by reversal cells stimulate bone formation and conclude bone resorption. *Formation phase*: mechanical signals and PTH curb sclerostin expression by osteocytes, resulting in Wnt signalling activation to promote osteoblast differentiation. *Termination phase*: osteocytes secrete sclerostin to terminate bone formation. The newly-formed bone is mineralized. Reprinted from Ref. [9].

Bone remodelling occurs due to the composite interactions of various cells in a closely-regulated environment. This process has five distinct and sequential phases (Figure 1).

1. Activation Phase: This is the first phase that requires a trigger generated either from cytokines released during apoptosis of osteocytes in the impaired bone, the detection of physical forces by osteocytes in the normal bone, or the hormonal (examples are estrogen or parathyroid hormone, PTH) actions on bone cells as a feedback mechanism for the systemic fluctuations due to homeostasis.

2. Resorption Phase: Osteoblasts react on the mechanical or endocrine signals via the engagement of preosteoclastic cells to the remodeling location. Cytokines, secreted by osteoblasts, promote the propagation of these precursor osteoclastic cells along with coordinating the differentiation to multinucleated mature osteoclasts. They further stimulate resorption of bones and prolong the lifespan for these mature osteoclasts. The mature cells anchor onto the bone surface and create an isolated microenvironment beneath the cells where dissolution of mineralized matrix occurs to create resorption lacunae.

3. Reversal Phase: Following osteoclast-mediated bone resorption, reversal cells remove the matrix debris before receiving or producing signals to initiate the transition from resorption to bone growth in BMUs. Studies have shown that the reversal cells express osteogenic markers, such as Runx 2, which are usually expressed by the cells of the osteoblast lineage [10]. Reversal cells modify or smoothen the bone surface resorbed by osteoclasts making it a suitable osteoblast maturation.

4. Formation Phase: Degraded bone matrix, osteoclasts, and possibly reversal cells create molecular cues to encourage the return of mesenchymal stem cells or premature osteoblast precursors to the resorption lacunae. These cells will then undergo differentiation and eventually mineralization to form new bone.

5. Termination Phase: After a comparable quantity of resorbed bone is restored with new mechanically stronger bone, the remodelling cycle concludes [9]. This bone resorption process occurs for weeks, whereas new bone formation takes months.

In the regular bone remodelling cycle, tight coupling of bone-forming osteoblasts and bone-resorbing osteoclasts occurs consecutively at a constant functional site without alteration in bone shape, bulk, or quality. Dysfunction of bone remodelling can cause the development of several metabolic bone disorders, including osteoporosis, osteopetrosis, and Paget’s disease [4].

Postmenopausal osteoporotic fractures have become a global concern. Globally, about 9 million people are affected by osteoporotic fractures per annum [11]. The suboptimal microenvironment of fragile osteoporotic bone hinders new bone formation [12]. The physiological imbalances cause bone resorption to exceed bone formation, which causes delayed fracture healing, hence, leading to post-surgical complications, such as implant loosening, mal-union, and implant failure [13,14,15]. Moreover, poor bone microarchitecture and low bone mineral density impair the anchorage and osseointegration of orthopaedic implants [16]. Revision surgeries impose huge physiological and economical burdens on elderly patients, not to mention the associated risks and complications. Anti-osteoporotic drugs were administered as an adjunct to surgery to reduce post-surgical complications. Systemic administration of anti-osteoporotic drugs, such as strontium ranelate and parathyroid hormone (PTH), augmented fracture healing in osteoporotic patients [17,18,19,20]. Bone-stimulating agents, such as bone morphogenetic protein-2 and -7, were also administered locally to stimulate bone healing and reduce post-surgical complications [21,22]. Additionally, technologies with new implant design, use of bone cements, new implant coatings have been explored to improve implant fixation in osteoporotic bone [23,24,25,26]. High bone resorption of osteoporotic bone is not desirable, especially bone resorption at the bone-implant interface may lead to non-union and implant failure. Understanding the physiology of osteoclastic bone resorption and influence of implants on osteoclast development is crucial to determine the success of the implant integration. The biology of osteoclasts and influence of various biomaterials on osteoclast development has been discussed in detail in the subsequent sections of this review.

### 1.2. Osteoclasts

**Figure 2:**
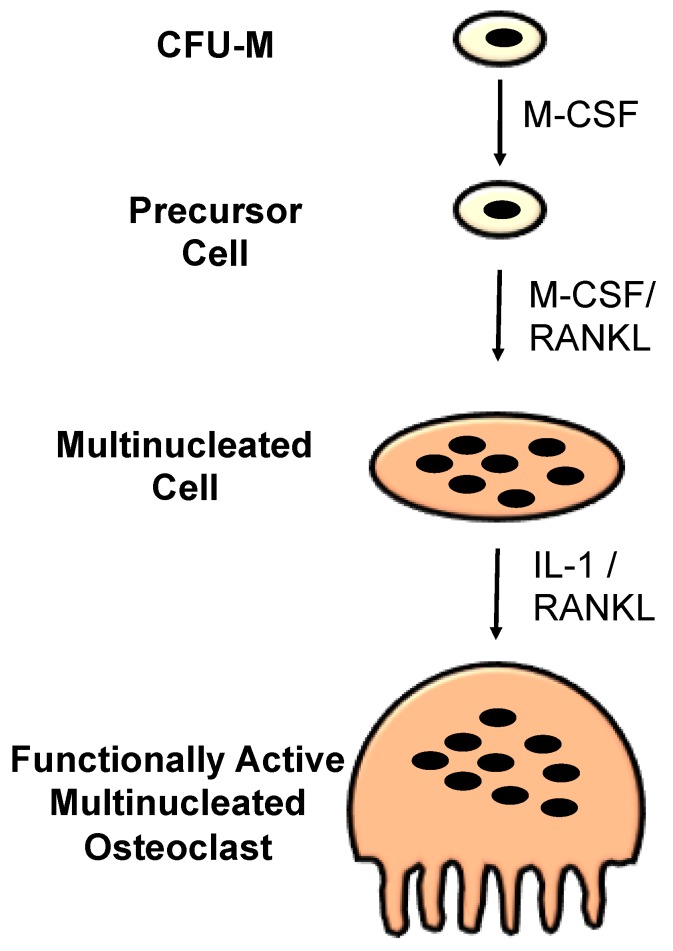
Schematic representation of osteoclast differentiation pathway and various cytokines involved at various stages of differentiation.

Osteoclasts are multi-nucleated macrophage-like cells, which originated from hematopoietic stem cells of monocyte-macrophage lineage. These cells are highly migratory and they are the only ones in charge of mineralized bone matrix resorption in remodelling. Osteoclastogenesis consists of several steps, including survival of progenitors from hematopoietic stem cells, differentiation of progenitors to mononuclear preosteoclasts, fusion into multi-nuclear mature osteoclasts, and activation to form bone-resorbing osteoclasts (Figure 2) [27].

Osteoclast differentiation and activation are affected by several cytokines and growth factors. Among them, RANKL and monocyte/macrophage colony–stimulating factor (M-CSF) are two essential factors [28,29]. RANKL is secreted through osteoblasts, osteocytes, and stromal cells, while M-CSF is produced by osteoblasts, stromal cells, and activated immune cells. Both RANKL and M-CSF, combining with receptors found on the monocytic/macrophagic cells, stimulate the undertaking to osteoclast phenotype; an activity restricted by osteoprotegerin (OPG) [27]. Additionally, recent studies have shown that tumour necrosis factor α (TNFα) [30], interleukin 1 (IL-1) [27], and lipopolysaccharides [31] can affect osteoclast development without RANKL-RANK interaction.

**Figure 3:**
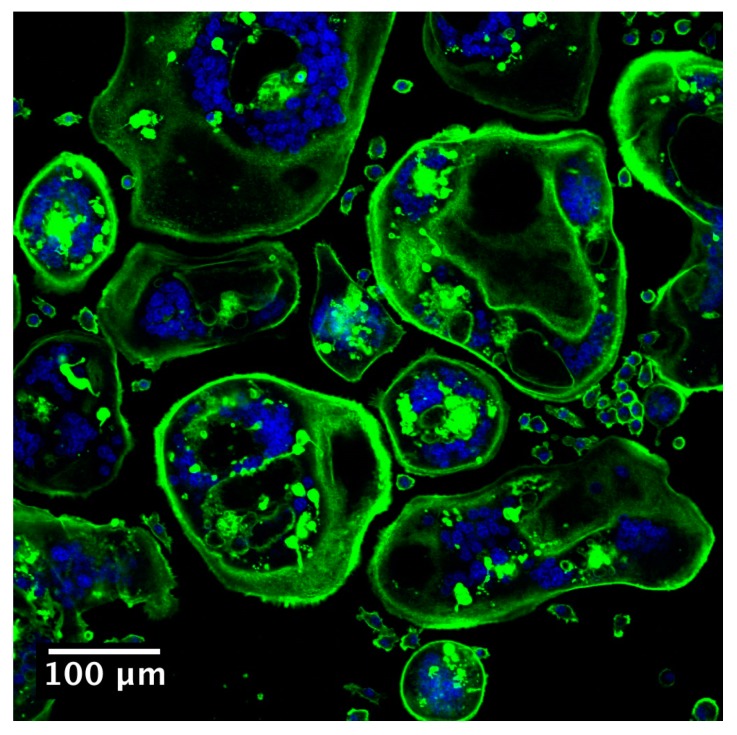
RAW 264.7 cells were cultured in presence of RANKL for five days, to induce osteoclast differentiation. The actin of multinucleated osteoclast cells is stained with phalloidin Alexa fluor 488 (green) and nuclei are stained with DAPI (blue).

During bone remodelling, osteoclasts first attach to bone surfaces through distinctive podosomes, adhesive networks that have actin filaments as the central core. They then undergo profound morphological changes and polarize to activate bone-resorbing cells [32]. Four different membrane domains can be perceived in activated osteoclasts: the sealing zone, the ruffled border, the basolateral domain, and the functional secretory domain [33]. Both the sealing zone and ruffled border are adjacent to the bone, while the basolateral domain and functional secretory domain are not. The sealing zone is a ring-like superstructure formed by rearrangement of podosomes upon activation of osteoclasts. It consists of a highly-ordered filamentous actin bundle that can be easily investigated with confocal microscopy after immunostaining with phalloidin (Figure 3).

This serves as a diffusion barrier. Through the sealing zone, osteoclasts tightly adhere to the bone surface and seal a closed compartment where bone resorption takes place. The ruffled border, which is away from the surrounding tissue due to the presence of sealing zone, forms part of the cell membrane.

The sealing zone and the ruffled border are characteristic structures for bone resorbing-osteoclasts. In the ruffled border, a vacuolar-type H+-ATPase pumps protons to help acidify the resorption lacuna (pH < 4) [34,35]. The mineral phase of bone is dissolved mainly due to the presence of local acidification. At the same time, several matrix metalloproteinases (MMPs), lysosomal proteinases, like cathepsin K [36,37], as well as additional cysteine proteinases are released [38]. These enzymes are responsible for degrading the organic phase of bone, primarily collagen I fibres. Tartrate-resistant acid phosphatase (TRAP) is secreted from the ruffled border [4], which is often used as a characteristic marker of osteoclasts [39]. The degraded products are subsequently endocytosed across the ruffled border and transcytosed in vesicles, and released through the functional secretory domain into the extracellular space. Excluding the sealing zone, ruffled border, and the functional secretory domain, the remaining membrane is known as the basolateral membrane. In the transcytosed vesicles, TRAP with highly-destructive reactive oxygen species can further implicate the degraded products [4,6,33].

### 1.3. In Vitro Models of Osteoclasts

In vitro experiments are often performed to investigate cell material interactions in the early phases of biomaterial development. Desirable conditions can be easily defined for cost effective screening of the potency in newly-developed biomaterial. The cell source must be identified when using osteoclast cultures. Both primary osteoclast precursor cells [40,41] and cell lines [42,43] are routinely used. Precursor cells are either harvested from bone marrow of animals/humans, or obtained from peripheral blood (mononuclear cells) [44]. One of the advantages in using precursor cells is that they possess high differentiation potency, and cytokines, for example, M-CSF and RANKL, are sufficient to differentiate these cells [45]. Although primary cells have a similar phenotype as cells in the body, they often have a limited lifespan and are genetically diverse if collected from different donors [46]. Additionally, primary osteoclasts are relatively scarce, as well as difficult to culture and study in vitro as compared to primary osteoblasts.

On the other hand, preosteoclasts obtained from cell lines are homogeneous with high differentiation potential [47]. The RAW 264.7 macrophage cell line of murine origin can differentiate into osteoclasts when stimulated with RANKL. It, along with another macrophage-like cell line, the C7 cell line, has been widely utilized for in vitro osteoclastic studies and bone remodelling [42,48]. Currently no human cell lines for osteoclasts have been established.

## 2. Biomaterials

Biomaterials have been widely used to restore the functions of damaged tissues in the human body. Currently, biomaterials with diverse chemical and physical properties are developed to suit the mechanical requirements and biological function of diverse tissue types, which vary from soft tissues, such as skin, to hard tissues such as bone [49,50,51]. In this review, the various biomaterials used in bone, such as metals, bioceramics, and polymers, has been reviewed. Each material type has its own advantages and disadvantages, as listed in Table 1. The success of a biomaterial depends on the host response and biological function of the implant. This is mainly dependent on the biomaterials’ interaction with the cells when exposed to the tissue environment in the human body. The surface physiochemical properties of the implant affect the cellular responses. The importance of the osteoclast response in the bone-implant interface has been recognized [52]. Yet, the response of osteoclasts on bone implants has not been as widely explored as osteoblasts. Hence, the review mainly focuses on the osteoclast activity on implants and studies of osteoclast response to different types of biomaterial is discussed in subsequent sections.

## 3. Metals

### 3.1. Introduction

Metals, like stainless steel, cobalt-chromium, titanium, and its alloys, have been widely used in fracture fixation and joint replacement for decades [53]. With the aging population and active lifestyle, the demand for metal implants are expected to increase in orthopaedic surgery [54]. Compared to bioceramics and polymeric biomaterials, metals are the first choice in load-bearing orthopaedic usage because of their outstanding mechanical properties. Among the metals, titanium and its alloys are by far the most prevalent, thanks to their exceptional chemical qualities and biocompatibility with human tissues [53].

Despite the prevalent use of metals, clinically, successful integration of metal implants with bone tissue, known as osseointegration, remains a concern. Osseointegration indicates the forming of a direct interface between the implant and bone, short of the soft tissue interfering [55,56]. High mechanical stability of implants in the host tissue can be obtained by having no foreign body response or encapsulation. At the bone-implant interface, constant dynamic remodelling requires close collaboration of osteoclasts and osteoblasts for osseointegration. Although much effort has been devoted to study the effects of different surface characteristics on metals in osteoblast bone formation, little data is presented about the osteoclast interaction with metal surfaces. Surface modification of metal implants is an encouraging advancement to improve osseointegration [56]. Surface topography and chemical modifications are commonly used to modulate cell behaviours on metal surfaces.

### 3.2. Surface Topography

Surface topography describes the general surface landscape involving undulations, pores, gradients, and small variations in an atomically flat surface to visible grooves and texture. Measuring arithmetical mean roughness (Ra) is one of the widely-used techniques to quantify surface roughness. Sandblasting, acid etching, and grinding are often used to increase surface roughness.

Several groups have studied osteoclast formation and activation on rough surfaces. Sommer et al. used titanium, TiAl6Mo7, CoCr28Mo6, and FeCrNi substrates with polished and sandblasted surfaces to evaluate osteoclastogenesis in vitro [57]. The results showed that TRAP activity and the number of osteoclast were higher on the sandblasted surfaces than that on the polished surfaces, while different metal alloys did not affect osteoclastogenesis significantly. Brinkmann et al. investigated the osteoclastogensis on titianium substrates with smooth (TS), acid-etched (TA), and sandblasted acid-etched (TLA) surfaces and compared it with that on the native bone using RAW 264.7 cell lines [58]. The results revealed that osteoclasts on rough surfaces such as TA and TLA showed similar osteoclastogenesis to those on native bone, whereas impaired osteoclastogenesis was observed on smooth surfaces. Nanotube structures on titanium surfaces by anodization has also been fabricated to investigate its interaction with osteoclasts [59]. Nanotube diameters of 30, 80, and 120 nm were prepared by anodization at 10, 40, and 60 V. The number of osteoclasts, TRAP activity, and the related gene expression on nanotube titanium were decreased compared to the pristine titanium. Osteoclastogenesis decreased with increasing the nanotube diameter.

Titanium surfaces were treated to varying roughness via wet grinding using 2000-, 1200-, 600-, and 180-grit abrasive papers. Molecular analysis of the initial osteoclast differentiation was then conducted [60]. It revealed that osteoclast differentiation markers, like TRAP and cathepsin K, increased in a roughness-dependent manner under RANKL stimulation. The gene expression of RANKL receptor, RANK, and its adapter protein TNF receptor-associated factor 6 (TRAF6) boosted with surface roughness. It was claimed that surface roughness assisted the maturation of osteoclasts by activating the RANK-TRAF6 pathway.

Surface topography can influence the stability and integrity of the sealing zone, which have imperative functions on bone resorption. By ranging the titanium roughness from 1 to 4.5 μm *R*_a_ to allow osteoclast bonding, Geblinger et al., investigated the sealing zone dynamics with direct live-cell microscopy [61]. Surface roughness stabilized the sealing zone, but negatively affected its translocation rate. Ridge-like barriers running parallel to the perimeter locally arrested the sealing zone. However, by advancing through less obstructed regions, the barriers could be bypassed. Nonetheless, when the sealing zone was surrounded by steep obstacles, the translocation would eventually stop and stabilize the sealing zone rings.

In general, increased osteoclastogensis are demonstrated on rough surfaces than those on smooth ones. Metal implants with rough surfaces have been shown to establish better osseointegration in clinical use [62]. It is speculated that poor osteoclast differentiation on smooth surfaces could be related to the delayed osseointegration of smooth surfaces compared to that of rough ones [58,63]. However, imbalanced osteoclast-osteoblast ratios due to elevated osteoclastogensis on rough surfaces remains a common concern.

### 3.3. Surface Chemistry

Surface chemical modification of metals with biomolecules and hydroxyapatite has been investigated for osteoclastogenesis. Makihira et al. covalently immobilized OPG-Fc fusion protein, a competitive inhibitor of RANKL, on titanium surfaces to improve osseointegration by preventing osteoclast formation locally [64]. The results showed that the gene expression for TRAP and cathepsin K of RAW 267.4 cells under RANKL stimulation were significantly reduced on OPG-Fc-immobilized titanium surfaces compared to that of the pristine surfaces. It is still unclear whether the immobilized OPG-Fc blocked RANKL-RANK interactions on the membrane. Moreover, this novel metal implants requires further in vivo animal models.

Decreased periprosthetic bone quality in osteoporotic patients inhibits implants from integrating with the surrounding bone. Surface coating with anti-osteoporotic drugs can impair bone resorption of osteoclasts around host bone tissues, which will benefit osteoporotic patients [65,66]. Bosco et al. developed a hydroxyapatite coating incorporated with bisphosphonate anti-osteoporotic drugs on titanium surfaces [67]. The electrospray deposition technique was used to deposit a hydroxyapatite coating without compromising the drug activity. Another interesting approach was developed by Huang et al. wherein polyelectrolyte multilayer coating techniques were used to immobilize anti-osteoporotic drug, calcitonin, and osteoinductive bone morphogenetic protein 2 (BMP-2) on a titanium surface [66]. Released calcitonin and BMP-2 from the titanium surface were expected to accelerate bone formation with a concomitant inhibition on bone resorption. In vivo results 30 days and 60 days after implantation for osteoporotic rabbits demonstrated that the coating on the implants enhanced bone-implant osseointegration significantly.

## 4. Bioceramics

### 4.1. Introduction

Bioceramics refer to biocompatible ceramic materials, such as crystalline and amorphous forms. They can be categorized according to their chemical composition into two groups: calcium phosphates (CaP), and the other constituting silicate-based bioactive glasses and glass-ceramics, like alumina and zirconia [68,69]. CaP ceramics include several materials with different chemical compositions and crystal phases. Among them, the two most studied CaP ceramics are beta-tricalcium phosphate (β-TCP) and hydroxyapatite (HA). Bioactive glasses and glasses-ceramics are made typically from the Na_2_O-CaO-MgO-P_2_O_5_-SiO_2_ system of varying composition. Bioactive ceramics, such as HA, β-TCP, and bioactive glasses, have been used as bone graft substitutes or scaffolds. Bioinert ceramics, including alumina and zirconia, have been used as parts in an integrated orthopaedic implant system during surgery. Some physical forms of bioceramics are granules, block, scaffolds, cements, and coating on metal implants.

Bone graft substitutes or scaffolds should be entirely degraded in a suitable time frame for effective bone regeneration. However, an optimal degradation rate is not defined for any biomaterial [7]. It is generally accepted that degradation of bioceramics is established on two distinctive mechanisms: resorption by chemical dissolution, passively, and cell-mediated degradation by osteoclastic resorption. Modulation on osteoclastic resorption of bioceramics is mainly through tuning the material compositions and surface topography.

### 4.2. Material Composition

β-TCP degrades too fast due to high solubility. On the other hand, HA is essentially insoluble and its degradation is only limited to the surface by osteoclasts since cells are not able to breach the microporous ceramic arrangement. Biphasic calcium phosphate (BCP), a mix of HA and β-TCP with varying ratios, may provide an optimal formulation in terms of bone resorption and final degradation. Yamada et al. investigated using neonatal rabbit bone cells to determine osteoclastic resorption on HA substrates, β-TCP substrates, and two types of BCP substrates in different HA/β-TCP ratios of 25/75 and 75/25 for two days [70]. The results showed that BCP with a HA/β-TCP ratio of 25/75 had the most extensive resorption by osteoclasts, which produced typical track-like resorption lacunae. On β-TCP substrates, smaller discontinuous island-like lacunae can be observed. No resorption lacunae can be found on HA or BCP with a HA/β-TCP ratio of 75/25. At the osteoclast-ceramic interface, it is speculated that free calcium ions in the acidic microenvironment may actually change the resorption pattern. In another study, Mayr et al. evaluated the degradation behaviour of HA, β-TCP, and four BCP with different HA/β-TCP ratios through stimulated osteoclast-like RAW 264.7 cells [71]. The monocytic progenitor cells were found to have matured into osteoclasts on all ceramics. The cell differentiation was the greatest on ceramics with HA/β-TCP ratios of 80/20, 60/40, and 40/60. All but β-TCP ceramic had bone resorption. It is claimed that BCP with HA/beta-TCP ratios of 80/20 and 60/40 revealed the most potential to assist as synthetic bone substitute materials due to their optimized dissolution and resorption properties. HA was resorbed by osteoclasts matured from rat bone marrow cells cultured after eight days and the U-937 human monocyte-like cell line after 21 days [72,73]. The contrast in results may be due to the different osteoclast cells used and culture time in studies.

Metals, like strontium (Sr), zinc (Zn), and magnesium (Mg), have been doped into bioceramics to influence osteoclast behavior [74,75,76]. Roy et al. assessed the outcomes of 1.0 wt % Sr and 1.0 wt % Mg incorporated into β-TCP on the osteoclast differentiation of RAW 264.7 cells under stimulation of RANKL [76]. Osteoclasts could be observed on β-TCP and Sr-incorporated β-TCP at day 8, but the cells were lacking on Mg-incorporated β-TCP. Zn-doped β-TCP (0.25 wt %) was reported to restrict osteoclast differentiation and a greater resorption pit volume was observed on this substrate after 28 days as in contrast with that of β-TCP [75]. Yamada et al. reported that Zn-doped β-TCP with a Zn content of 0.316 wt % and 0.633 wt % in β-TCP subjugated osteoclastic resorption. This would be attributed to the inhibition of osteoclast resorbing activity, which was demonstrated by fewer actin rings formed on Zn-doped β-TCP than those on β-TCP [77]. Recently, Sr has also been added into CaP cement and bioactive glasses to reduce the resorption [74,78]. Schumacher et al. found that Sr in CaP cement significantly decreased osteoclastic resorption in comparison to the pristine CaP cement, but did not inhibit osteoclastogenesis [78].

Silicon (Si) has also been incorporated into HA and CaP cement [79,80,81,82]. Botelho et al. demonstrated higher osteoclastic resorption activity on 1.5 wt % Si-substituted HA compared to that of HA [81]. In another study, osteoclast numbers after 21 days in vitro were comparable on HA, Si-HA (0.5 wt % Si) and Si-HA (1.2 wt % Si), but actin ring sealing zone morphology on Si-HA bore semblance to those found normally on bone. Greater individual pit volumes detected on Si-HA (1.2 wt % Si) may result from the relatively constant sealing zones of osteoclasts and improved lattice solubility [79]. Si increased osteoclast activity in vitro after doped into CaP cement [82]. However, Si released from S53P4 bioactive glass inhibited osteoclastogenesis [83], while another report depicted the presence of resorption lacunae on sol–gel-derived bioactive glasses and glass ceramics [84,85].

### 4.3. Surface Topography

In addition to material composition, the surface topography can be tailored to affect osteoclast behavior on bioceramics. Costa-Rodrigues assessed the osteoclastogenesis in human peripheral blood mononuclear cells (PBMCs) together with the co-culture of human bone marrow cells (hBMCs) on surface-abraded HA substrates of three different roughnesses (*R*_a_ from 0.0437 to 0.582 μm). Characteristic osteoclast properties were seen in PBMCs added with M-CSF and RANKL, or the co-culture with hBMCs. Osteoclastogenesis improved with greater surface roughness in PBMCs added with M-CSF and RANKL, but decreased when in co-culture with hBMCs [86]. HA substrates of three distinct surface roughness (*R*_a_ 0.02, 0.18, and 1.45 μm) were also used to investigate osteoclastgenesis with stimulated RAW 264.7 cells [7]. It was shown that the HA surface with a *R*_a_ of 0.18 μm demonstrated the greatest osteoclastic differentiation, indicating the positive effect of smaller structures. Conversely, a smoother surface encouraged greater osteoclast formation than one with a rough surface, which could be rationalized by the possibility of optimal cell spreading on the rough surface. Higher resorption activity of human-derived osteoclasts on rough HA thermal spray coating is observed versus those on polished HA coating of metal implants [87]. Another study using neonatal rabbit osteoclasts showed the manifestation of resorption lacunae only on smooth HA coatings, while micro-rough surfaces reduced such lacunae [88]. It can be shown that the differences in differentiation routes of pre-osteoclasts can have a significant effect on osteoclastogenesis for HA surfaces with different roughness.

By modifying surface architecture, it is very probable to regulate the osteoclastic resorption of β-TCP. β-TCP substrates with either submicron- or micron-scale surface topographical structures were used to investigate resorption by human peripheral blood monocyte-derived osteoclasts. On submicrostructured β-TCP, osteoclasts considerably resorbed the substrate, whereas microstructured β-TCP caused the attenuation of osteoclast survival, TRAP activation, and fusion. Effectively, no actin ring and resorption was seen on microstructured β-TCP. This approach presented an advantageous approach in designing resorbable substitutes for bone grafts [89].

## 5. Polymers

Interaction of polymeric biomaterials with osteoclasts has been investigated limitedly, although polymers, especially biodegradable ones, are often used to fabricate scaffolds in bone tissue engineering and regeneration medicine. Recently, chitosan, a natural polymer from crustaceans, was evaluated for osteoclast formation before and after fibrinogen (Fg) modification with primary human peripheral blood monocytes [90]. Similar cell adhesion patterns were observed in both unmodified and Fg-modified chitosan substrates. By 21 days of culture, Fg-modified chitosan has a significantly higher number of multinucleated osteoclasts (>10 nuclei/cell) than that of the unmodified one. Quantification of cell-induced material resorption showed that Fg modification resulted in considerably greater resorption by 17 days of culture. Textile chitosan fibre scaffolds with or without collagen I, which is widely used as a scaffold for bone regeneration, were investigated on their interactions with mature osteoclasts derived from human primary monocytes after adding M-CSF and RANKL [91]. The results revealed that both modified and unmodified chitosan fibre scaffolds supported adherence and maturation of monocytes to osteoclasts. The capability of textile chitosan fibre scaffolds to encourage the migration and bonding of human monocytes before merging to form multinucleated osteoclast cells was also exhibited in the study. Interestingly, chitosan had also been added into CaP cement to inhibit the osteoclastic resorption [92]. Collagen coating on polymers can further enhance osteoclastogenesis. When polydimethylsiloxane (PDMS) was coated with collagen, increased osteoclastic activity was reported [93]. Yet, clumps of undifferentiated RAW 264.7 cells were observed at higher surface densities of collagen on PDMS. This suggested that substrate compositions affected the behaviour of monocytes.

Synthetic biodegradable polymer, poly(3-hydroxybutyrate-*co*-3-hydroxyvalerate) (PHBV), reinforced with various phases of calcium phosphate allowed osteoclast attachment although it was unsuccessful in materializing actin rings or resorption pits on every PHBV surfaces [94]. Similar results were observed in another synthetic biodegradable polymer, poly(l-lactic acid) (PLLA) [95].

## 6. Conclusions and Outlook

Less attention is spent on understanding the influence of orthopaedic biomaterials on osteoclast development than on osteoblasts. The successful clinical use of orthopaedic biomaterials requires the balanced activation of osteoclasts and osteoblasts, as with that in native bone remodelling [96]. Understanding the mechanism of osteoclast interactions with orthopaedic implant would be beneficial to design suitable metal implant surface, and engineer appropriate scaffolds which can then modulate osteoclast development in desirable ways. Research attention in this area is crucial.

Metals are widely used in clinical settings to restore the mechanical strength and function of the damaged bone. The physical and chemical properties of the implant surface can be modified to control the osteoclast development. For instance, surface roughness enhanced osteoclastogenesis, whereas chemical modification of the implant surface with proteins or anti-osteoporotic drugs decreased osteoclast function. Bioceramics researched as bone graft substitutes or scaffolds were shown to influence osteoclast development. The osteoclast development varied with different chemical composition of bioceramics; for instance, Sr incorporation reduced osteoclastic resorptive activity, whereas Si increased osteoclast activity. The surface topography of bioceramics also controlled osteoclast differentiation. Like bioceramics, polymers are used to develop bone scaffolds in bone tissue engineering. Although research of the osteoclast-polymer response is limited, the studies reviewed here depict the influence of different polymers on osteoclast response. This shows that the choice of polymer affects the osteoclast activity and, hence, can be tailored to meet the clinical needs.

Fractured bone at the submicron level has a topographically complex surface, which provides adequate biochemical and biophysical cues to osteoblasts and osteoclasts for their proliferation and differentiation, for forming healthy bone. Understanding the natural surface topography and chemistry of the bone’s surface, osteoclast resorption pits and trails could provide valuable information. Simulating such natural surface features on implants via various surface modification techniques would induce osteogenesis in a way comparable to natural bone remodelling. Such biomimetic approaches will help to enhance osseointegration of implants. Additionally, the osteoclast interactions with biodegradable polymers and bioceramics have been reviewed to a small extent, although they are widely used as scaffolds in bone tissue engineering. Detailed investigation of osteoclast-induced bioresorption of scaffolds will help to optimize the design of scaffolds.

Given that the osteoclasts and osteoblasts are in complex crosstalk in the in vivo environment, co-culture of osteoclasts and osteoblasts will provide a more suitable model to investigate the nature of bone remodelling on orthopaedic biomaterials. However, challenges still exist to design optimal culture medium compositions, define the osteoclast/osteoblast ratio, and culture time to stimulate the native bone environment. Nonetheless, separate cultures of osteoblasts and osteoclasts help to understand the independent physiologies of their development, which is one of the advantages of using independent cell cultures over co-cultures.

## Figures and Tables

**Table 1:** Advantages and disadvantages of orthopaedic biomaterials.

Biomaterials	Advantages	Disadvantages
Metals	High strength, fatigue resistance	Metal ion toxicity, wear
Bioceramics	High bioactivity (bioactive glasses), biodegradability (TCP), low friction coefficient and wear rate (bioinert ceramics)	Brittleness, low fatigue resistance
Polymers	Ease of ease of manufacture and modification	Low strength
